# Prevalence of post-traumatic stress disorder and its association with adverse childhood experiences among refugee and host-community children and adolescents in Jordan

**DOI:** 10.3389/frcha.2025.1648195

**Published:** 2026-01-12

**Authors:** Sara Abu Khudair, Yousef Khader, Osama Al Kouri, Mohannad Al Nsour, Eizaburo Tanaka

**Affiliations:** 1 Eastern Mediterranean Public Health Network (EMPHNET), Amman, Jordan; 2 Department of Community Medicine, Public Health and Family Medicine, Faculty of Medicine, Jordan University of Science and Technology, Irbid, Jordan; 3 Faculty of Nursing, Yarmouk University, Irbid, Jordan; 4 Center for Research on Counseling and Support Services, The University of Tokyo, Tokyo, Japan

**Keywords:** adolescent, adverse childhood experiences, child, Jordan, mental health, post-traumatic stress disorder, refugees

## Abstract

**Background:**

Exposure to adverse childhood experiences (ACEs) is linked to the development of various psychological disorders, particularly post-traumatic stress disorder (PTSD). This study aimed to determine the prevalence rates of ACEs and high PTSD symptoms and to investigate their association among children and adolescents in Jordan.

**Methods:**

A large-scale school-based national survey was conducted among children and adolescents in the host and refugee populations aged 8–18 years in Jordan, utilizing a multi-stage stratified cluster sampling technique. The study questionnaires contained standardized and psychometrically validated instruments to assess ACEs and PTSD.

**Results:**

A total of 8,000 children and adolescents were included. The most prevalent ACEs among children were COVID-19 infection (43.2%), food insecurity or inadequate clothing (22.1%), and exam failure (21.7%), while for adolescents, COVID-19 infection (60.3%), exam failure (43.2%), and the death of a family member (31.7%). High PTSD symptoms were observed in 31.4%, more prevalent in females than males (33.0% vs. 29.3%, *p* = 0.003) and adolescents than children (35.5% vs. 24.6%, *p* < 0.001). For both children and adolescents, feeling unloved by family was significantly associated with high PTSD symptoms (OR = 2.1, *p* < 0.001). Other significant ACEs included death of a family member (children: OR = 1.9; adolescents: OR = 1.8) and food insecurity/inadequate clothing (OR = 1.7 for both). In children, exam failure was significant (OR = 1.5), while in adolescents, it included physical assault (OR = 1.4), COVID-19 infection (OR = 1.3), serious accidents (OR = 1.3), and emotional abuse (OR = 1.6). Experiencing ≥4 ACEs greatly increased PTSD odds (children: OR = 5.0, adolescents: OR = 4.3; *p* < 0.001).

**Conclusion:**

Findings highlight a high prevalence of PTSD among children and adolescents exposed to ACEs, with multiple ACEs linked to higher PTSD symptoms and a cumulative pattern further increasing this risk. Results underscore the need for targeted interventions addressing key ACEs by age group and the development of comprehensive prevention programs involving parents, families, schools, communities, and broader society. Interventions should also aim to mitigate the cumulative impact of multiple ACEs and address them collectively rather than individually.

## Introduction

Adverse childhood experiences (ACEs) are potentially traumatic events occurring before the age of 18, including physical, emotional, and sexual abuse, physical and emotional neglect, and household dysfunction, including domestic violence, substance abuse, mental illness, parental separation, and incarceration (1, 2). Exposure to ACEs is linked to the development of various psychological disorders, particularly post-traumatic stress disorder (PTSD) (3, 4). In a study involving young girls, a gradual increase in the likelihood of probable PTSD was observed with higher ACE exposure levels, with a single ACE exposure such as firearm abuse or rape, increasing the likelihood of probable PTSD nearly fivefold (5).

Symptoms of PTSD in children and adolescents include reliving the trauma through intrusive thoughts and flashbacks, avoiding trauma-related triggers, experiencing negative mood shifts, and heightened arousal and reactivity, which can hinder a child's development, influencing their emotional, cognitive, and social progress (6). PTSD in children is linked to depression, aggression, academic struggles, and social challenges (7, 8). Also, the prolonged psychological distress associated with PTSD can increase the likelihood of developing chronic diseases and conditions such as hypertension, hyperlipidemia, obesity, and cardiovascular disease (9, 10).

Recent findings from a nationwide survey highlighted major mental health and psychosocial challenges among children and adolescents in Jordan (11). In general, Jordan faces a combination of challenges including regional instability, a large influx of refugees, economic hardship, specific cultural norms surrounding mental health, such as stigma and limited mental health resources. This creates a complex environment in Jordan that heightens the risk of negative mental health outcomes, including ACEs and PTSD, among children and adolescents, particularly among refugee populations (12). A comprehensive study from Jordan revealed a high prevalence of child abuse and its negative consequences, coupled with a lack of awareness about child abuse and its impacts (13).

Despite PTSD's profound impact on children, most studies on ACEs and PTSD lack representative samples and few focus on low-income countries (14, 15). In Jordan, few studies have examined PTSD and its relationship with ACEs, particularly using a representative sample that includes a broad age range encompassing both children and adolescents (16). Additionally, children and adolescents form a large share of Jordan's population, and Jordan hosts one of the world's largest refugee populations relative to its size (17, 18). Also, both refugee and host-community youth in Jordan face significant stressors. Refugee children experience displacement, loss, and instability, while host-community children often face overcrowded schools and strained services (19, 20). These overlapping pressures increase vulnerability in both groups. Therefore, this study aimed to determine the prevalence rates of ACEs and high PTSD symptoms and to investigate the association between ACE and PTSD among children and adolescents living in Jordan. This study is crucial to inform the development of interventions and prevention policies specifically tailored to this population. Findings are important for mental health practitioners, educators, policymakers, and caregivers, enabling them to provide effective support and minimize the enduring effects of trauma.

## Methods

### Study design, sampling

The detailed methods of this large-scale national school-based cross-sectional survey conducted among children and adolescents in Jordan, including sample selection, data collection protocols, and the translation process, are thoroughly described in another paper (11). In summary, the survey aimed to capture a nationally representative sample, including Jordanian children and adolescents as well as Syrian and Palestinian refugees enrolled in grades 3–12. A multi-stage stratified cluster sampling technique was employed. Schools were selected from different governing authorities, including the Ministry of Education (MoE), private schools, and UNRWA schools for Palestinian refugees. Stratification was based on region (North, Central, and South) and governorate. Special attention was given to refugee contexts, including both regular MoE schools and Syrian second-shift schools, further stratified by gender. Students who had dropped out of formal schooling were included through non-formal education centers, with data collected from centers in the northern and central regions of Jordan, where most students enrolled in non-formal education programs are located. The sample of non-formal education centers was also gender-balanced.

### Data collection

Data collection took place between December 2022 and April 2023, during which trained data collectors visited schools and non-formal education centers. A pilot test was conducted in two schools in northern Jordan to evaluate the survey content and data collection process, and the researchers conducted regular monitoring of data quality. The study followed ethical guidelines, with informed consent obtained from caregivers and adolescents.

### Study instruments

The survey included two versions: a proxy parent version for parents of students in grades 3–6 and a self-report version for students in grades 7–12. The expected age of students in the parent-report version ranged from 8 to 11 years, and 12 to 18 years for the self-report version, according to the MoE educational ladder. Students in non-formal education programs completed the self-report version, as they were 12 years of age or older and therefore fit the self-report category of the study.

For instruments available only as self-report versions, questions were reworded by the research team to reflect parents’ responses about their children. Permission to use all tools was obtained from their respective developers. When an Arabic version was not available, forward–backward translation procedures were followed for both versions. The translated versions were then piloted within the target population. Modifications were minimal and focused primarily on enhancing clarity. Cronbach's alpha, measuring internal reliability, for the Children's Revised Impact of Event Scale (CRIES-13), used to assess post-traumatic stress disorder (PTSD) symptoms, was 0.876 for children and 0.883 for adolescents, indicating strong internal consistency. The mean inter-item correlation was 0.356 for children and 0.367 for adolescents, both within acceptable ranges.

#### Children's and adolescents’ characteristics

A total of 32 variables were used to capture the characteristics of the children and adolescents, including school characteristics, demographics, parental demographics, medical history (COVID-19 infection history, chronic diseases, special needs, regular medication use, mental health problems and family history of mental health issues), insurance status, length of stay for Syrian refugees, and socioeconomic status (SES).

### Adverse childhood experience (ACE)

Adverse childhood experiences were assessed using the Adverse Childhood Experiences (ACEs) Questionnaire and the Life Events Checklist for DSM-5 (LEC-5). The ACEs questionnaire is a 10-item instrument derived from the CDC–Kaiser Permanente ACEs Study (21). The list was revised and adapted to exclude sensitive questions such as sexual abuse; the final version consisted of four items assessing physical abuse, verbal abuse, physical neglect, and emotional neglect. The LEC-5 is a self-report measure designed to screen for potentially traumatic events across the lifespan (22), consisting of 16 events known to be associated with PTSD and one optional item assessing any other extraordinarily stressful event. Eleven LEC-5 items were included in the study tool to complement the ACEs questionnaire, along with three additional items related to exam failure and COVID-19. Responses were limited to two choices: “happened to me” and “did not happen to me”.

#### The children's impact of event scale-13 (CRIES -13)

PTSD symptoms were assessed using the CRIES-13, which includes 13 items measuring childhood PTSD symptoms (23, 24). It comprises an overall score and three subscales: intrusion, avoidance, and arousal. A cut-off score of 30 has been shown to be effective for screening for PTSD (23) and was used to categorize those with high PTSD symptoms. Higher scores indicate higher symptom severity.

The study questionnaire incorporated conditional branching for the ACEs and PTSD sections. Only those who reported experiencing at least one ACE proceeded to the ACE section to identify the most distressing ACE they or their children had encountered. They were then eligible to complete the CRIES-13 section.

### Statistical analysis

Data analysis was conducted using IBM SPSS 24 (IBM Corp., 2016). Frequencies and percentages were used to describe categorical variables. Chi-square tests were used to compare the prevalence of ACEs between children and adolescents, examine differences in PTSD categories across groups, and to compare PTSD prevalence according to ACE exposure. Multivariate binary logistic regression was used to analyze the association between PTSD and ACEs. The model included all studied ACEs, including non-significant ones, to allow a comprehensive evaluation of all potential contributors to PTSD. Retaining non-significant ACEs minimizes bias and ensures the model reflects the full range of experiences. The effects of ACEs were adjusted for gender and chronic illness, as both were significant in the model. Other socio-demographic variables were not significantly associated with PTSD and were excluded. A *p*-value of <0.05 was considered statistically significant.

## Results

### Participants characteristics

A total of 8,000 children and adolescents aged 8–18 years were included, with 42.9% boys and 57.1% girls. Of these, 44.9% were children (8–11 years), and 55.1% were adolescents (12–18 years). Most participants (64.2%) attended public schools, and the majority lived in the central (46.0%) and northern (42.3%) regions of Jordan, with 11.7% residing in the southern region. Jordanians were the largest group, making up 57.8% of children and 66.2% of adolescents, followed by Syrians living outside refugee camps. About 77.5% of participants reported their fathers were employed, while 22.6% reported their mothers were working. Around half of the participants belonged to medium-affluent families, and 22.2% had a confirmed COVID-19 infection.

### The adverse childhood experiences

Table 1 shows the prevalence rates of ACEs among children and adolescents. The most prevalent ACEs among children were being infected or having a family member infected with COVID-19 (43.2%), food insecurity or inadequate clothing (22.1%), and exam failure (21.7%), while for adolescents, being infected or having a family member infected with COVID-19 (60.3%), exam failure (43.2%), and the sudden accidental death of a family member (31.7%). Almost all ACEs were significantly more common among adolescents compared to children.

**Table 1:** The prevalence of adverse childhood experiences among children and adolescents.

Event	Children (8–11 years) *n* = 3,593		Adolescents (12–18 years) *n* = 4,407		Total *N* = 8,000	
	*n*	%	*n*	%	*N*	%
Infected or a family member infected with COVID-19	1,530	43.2	2,611	60.3	4,141	52.6
Failing an exam	772	21.7	1,878	43.2	2,650	33.5
The sudden accidental death of any of the family members	540	15.2	1,373	31.7	1,913	24.3
Physical assault (being attacked, hit, slapped, kicked, beaten up)	528	14.9	1,004	23.2	1,532	19.4
Insulted or belittled by a parent or other adult in the house	526	14.8	992	22.9	1,518	19.3
Car Accident	282	7.9	1,215	28.0	1,497	19.0
Did not have enough to eat, or had to wear old clothes	784	22.1	654	15.1	1,438	18.2
Feeling that no one in the family loves you/your child	310	8.7	953	22.0	1,263	16.0
Severely beaten by a parent or other adult in the house	234	6.6	694	16.1	928	11.8
Serious accidents at home, school, or during recreational activity	209	5.9	674	15.5	883	11.2
Fire or explosion	207	5.8	670	15.4	877	11.1
Combat or exposure to a warzone	198	5.6	542	12.5	740	9.4
Serious injury or harm you/your child caused to someone else	102	2.9	615	14.3	717	9.2
A family member died from COVID-19	166	4.7	454	10.5	620	7.9
Life-threatening illness or injury	126	3.5	489	11.3	615	7.8
Natural disasters (floods, intense streams of water, or earthquakes)	95	2.7	472	10.8	567	7.2
Exposure to toxic substances, such as dangerous chemicals	83	2.3	361	8.3	444	5.6
Assault with a weapon (being shot, stabbed, or threatened with a knife, or gun)	39	1.1	273	6.3	312	3.9

All differences between children (8–11 years) and adolescents (12–18 years) were statistically significant (*p* < 0.001).

### Prevalence of high levels of PTSD symptoms

**Figure 1:**
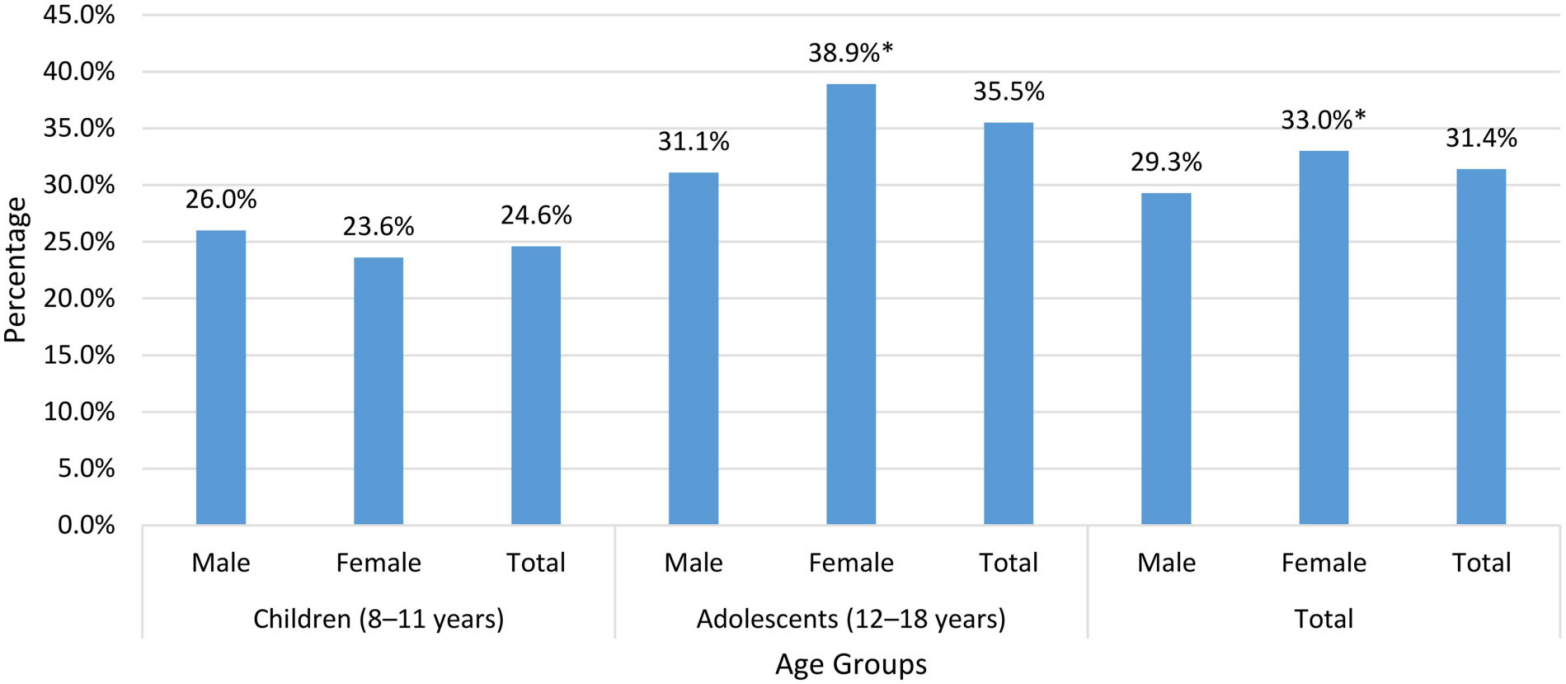
The prevalence of high levels of post-traumatic stress disorder symptoms among children and adolescents by gender and age group. * Indicates statistically significant gender difference.

About one-third (31.4%) of children and adolescents exhibited high levels of PTSD symptoms, higher in females than males in the total sample (33.0% vs. 29.3%, *p* = 0.003) and adolescents (38.9% vs. 31.1%, *p* < 0.001). Also, rates were higher in adolescents than in children (35.5% vs. 24.6%, *p* < 0.001), as shown in Figure 1. For children, the prevalence of high PTSD symptoms differed significantly according to nationality and was highest among Syrians living outside camps (30.2% vs. 21.5% among Jordanians, *p* < 0.001). The lowest prevalence was observed among Palestinians living in a camp (20.0%). Jordanians and Syrians living in camps had prevalence rates of 21.5% and 27.4%, respectively. Among adolescents aged 12–18 years, no significant differences in PTSD prevalence were observed across nationality groups. The highest prevalence is found among Palestinians living in a camp (37.7%), followed by Syrians living in camps (36.4%), Jordanians (35.9%), and Syrians living outside camps (33.8%), without a significant difference (*p* = 0.531). There was no statistically significant association between nationality and PTSD status in the total sample.

### Prevalence of high levels of PTSD according to adverse childhood experiences

The prevalence of high levels of PTSD symptoms among both children and adolescent groups was significantly higher for those who experienced any one of 12 adverse events out of the 18 events studied (Table 2).

**Table 2:** Prevalence of high levels of post-traumatic stress disorder symptoms according to adverse childhood experiences.

ACE exposure	PTSD							
	Children (8–11 years) *n* = 3,593				Adolescents (12–18 years) *n* = 4,407			
	*N*	*n*	%	*p*-value	*N*	*n*	%	*p*-value
Natural disasters (floods, intense streams of water, earthquakes)								
No	2,082	506	24.3	0.088	3,125	1,111	35.6	0.910
Yes	90	29	32.2		455	163	35.8	
Fire or explosion								
No	1,970	459	23.3	<0.001	2,948	991	33.6	<0.001
Yes	199	75	37.7		638	287	45.0	
Car accident								
No	1,903	461	24.2	0.297	2,409	879	36.5	0.116
Yes	265	72	27.2		1,157	391	33.8	
Serious accident at home, school, or during recreational activity								
No	1,980	471	23.8	0.002	2,927	993	33.9	<0.001
Yes	195	66	33.8		647	283	43.7	
Exposure to toxic substances such as dangerous chemicals								
No	2,088	508	24.3	0.475	3,216	1,139	35.4	0.384
Yes	79	22	27.8		355	134	37.7	
Physical assault (being attacked, hit, slapped, kicked, beaten up)								
No	1,708	365	21.4	<0.001	2,576	809	31.4	<0.001
Yes	464	169	36.4		984	460	46.7	
Assault with a weapon (being shot, stabbed, or threatened with a knife, or gun)								
No	2,134	525	24.6	0.538	3,303	1,170	35.4	0.266
Yes	38	11	28.9		268	104	38.8	
Combat or exposure to a warzone								
No	1,992	462	23.2	<0.001	3,040	1,039	34.2	<0.001
Yes	180	72	40.0		523	230	44.0	
Life-threatening illness or injury								
No	2,042	488	23.9	<0.001	3,085	1,055	34.2	<0.001
Yes	122	45	36.9		479	210	43.8	
The sudden accidental death of any of the family members								
No	1,672	363	21.7	<0.001	2,255	685	30.4	<0.001
Yes	489	172	35.2		1,312	583	44.4	
Infected or a family member infected with COVID-19								
No	930	246	26.5	0.047	1,112	347	31.2	<0.001
Yes	1,231	280	22.7		2,449	920	37.6	
A family member died from COVID-19								
No	2,005	479	23.9	0.011	3,110	1,100	35.4	0.430
Yes	158	52	32.9		437	163	37.3	
Serious injury or harm you caused to someone else								
No	2,072	504	24.3	0.151	2,923	1,020	34.9	0.012
Yes	94	29	30.9		595	240	40.3	
Insulted or belittled by a parent or other adult in the house								
No	1,692	360	21.3	<0.001	2,607	774	29.7	<0.001
Yes	473	171	36.2		952	502	52.7	
Severely beaten by a parent or other adult in the house								
No	1,956	441	22.5	<0.001	2,863	936	32.7	<0.001
Yes	216	94	43.5		682	333	48.8	
Feeling that no one in your family loves you								
No	1,884	406	21.5	<0.001	2,630	777	29.5	<0.001
Yes	279	124	44.4		932	499	53.5	
Did not have enough to eat, or had to wear old clothes								
No	1,455	281	19.3	<0.001	2,933	966	32.9	<0.001
Yes	708	252	35.6		635	310	48.8	
Failing an exam								
No	1,486	318	21.4	<0.001	1,802	567	31.5	<0.001
Yes	686	218	31.8		1,777	714	40.2	

### Multivariate analysis of the association between PTSD and adverse childhood experiences

For both children and adolescents, the child/adolescent's feeling of not being beloved by the family was significantly associated with the highest odds of PTSD (OR = 2.1, *p* < 0.001). For both age groups, the following ACEs were significantly associated with increased odds of high PTSD symptom levels: the sudden accidental death of any of the family members (OR = 1.9 for children and 1.8 for adolescents, *p* < 0.001), not having enough to eat, or having to wear old clothes (OR = 1.7, *p* < 0.001). Additionally, failing an exam was associated with high PTSD symptoms in children only (OR = 1.5, *p* < 0.001), while experiencing physical assault (OR = 1.4, *p* = 0.002), being infected or a family member infected with COVID-19 (OR = 1.3, *p* = 0.001), a serious accident at home, or school or during recreational activity (OR = 1.3, *p* = 0.021), and being Insulted or belittled by a parent or other adult in the house (OR = 1.6, *p* < 0.001) was associated with increased odds of high PTSD symptoms in adolescents only, as shown in Table 3.

**Table 3:** Multivariate analysis of the association between high post-traumatic stress disorder symptom levels and adverse childhood experiences*.

Event	High PTSD symptoms							
	Children (8–11 years) *n* = 3,593				Adolescents (12–18 years) *n* = 4,407			
	OR	95% confidence interval		*p*-value	OR	95% confidence interval		*p*-value
Feeling that no one in your family loves you	2.1	1.5	2.9	<0.001	2.1	1.7	2.6	<0.001
The sudden accidental death of any of the family members	1.9	1.5	2.5	<0.001	1.8	1.5	2.1	<0.001
Did not have enough to eat, or had to wear old clothes	1.7	1.4	2.2	<0.001	1.7	1.4	2.1	<0.001
Physical assault (being attacked, hit, slapped, kicked, beaten up)	1.3	1.0	1.8	0.043	1.4	1.1	1.6	0.002
Failing an exam	1.5	1.2	1.9	0.001	1.2	1.0	1.4	0.056
Assault with a weapon (being shot, stabbed, or threatened with a knife, or gun)	0.3	0.1	0.8	0.013	0.8	0.6	1.2	0.339
Infected or a family member infected with COVID-19	1.0	0.8	1.2	0.712	1.3	1.1	1.6	0.001
Serious accidents at home, school, or during recreational activity	1.2	0.8	1.8	0.364	1.3	1.0	1.6	0.021
Car Accident	1.0	0.7	1.4	0.911	0.8	0.7	1.0	0.018
Insulted or belittled by a parent or other adult in the house	1.2	0.9	1.6	0.346	1.6	1.3	2.0	<0.001
Exposure to toxic substances, such as dangerous chemicals	0.9	0.4	1.7	0.682	0.9	0.6	1.2	0.301
Natural disasters (floods, intense streams of water, earthquakes)	0.7	0.4	1.4	0.323	0.9	0.7	1.2	0.382
Fire or explosion	1.4	1.0	2.1	0.087	1.2	1.0	1.5	0.052
Combat or exposure to a warzone	1.4	1.0	2.1	0.070	1.2	0.9	1.6	0.123
Life-threatening illness or injury	1.4	0.9	2.2	0.185	1.2	0.9	1.5	0.207
A family member died from COVID-19	1.1	0.7	1.6	0.804	0.8	0.6	1.0	0.052
Serious injury or harm you caused to someone else	0.8	0.4	1.5	0.494	0.9	0.7	1.1	0.319
Severely beaten by a parent or other adult in the house	1.4	1.0	2.1	0.064	1.1	0.9	1.4	0.298

*Adjusted for gender and having chronic illnesses. The value of Nagelkerke R Square is 14.0% for children and 18.0% for adolescents.

### Number of ACE and PTSD symptoms

Across the sample, differences in the number of ACEs were more pronounced among children, with higher ACE counts more frequently reported among Syrian children, especially those living outside camps. In contrast, these disparities became less marked in the adolescent group, where the proportions of ACE exposure were more comparable across nationalities. Among children, significant differences in ACE exposure numbers were identified across nationalities (*p* < 0.001). For 0 ACEs, Syrians living in camps had a significantly higher proportion than Jordanians (34.6% vs. 24.6%, *p* < 0.001), and Syrians living outside camps (34.6% vs. 26.7%, *p* = 0.010). For 1 ACE, Jordanians had a significantly higher observed proportion compared with Syrians living outside camps (29.8% vs. 22.1%, *p* = 0.001). However, for the 4 or more ACEs, Syrians living outside camps had a significantly higher proportion than both Jordanians (20.9% vs. 15.3%, *p* = 0.002) and Syrians living in camps (20.9% vs. 14.5%, *p* = 0.018). Among adolescents, although the overall chi-square test was statistically significant, the proportions across most ACE categories were largely comparable across nationalities. The only significant difference appeared in the no ACEs category, where Syrians living in camps had a significantly higher proportion compared with Jordanian adolescents (17.9% vs. 11.1%, *p* < 0.001) and those of other nationalities.

An increased number of ACEs was significantly associated with higher odds of PTSD symptoms after adjusting for gender and chronic medical conditions. Experiencing four or more ACEs was linked to significantly increased odds of PTSD in children (OR = 5.0, 95% CI: 3.7–6.7, *p* < 0.001) and adolescents (OR = 4.3, 95% CI: 3.4–5.5, *p* < 0.001) (Table 4).

**Table 4:** The association between the number of adverse childhood experiences and high post-traumatic stress disorder symptom levels.

Number of events	High PTSD symptoms							
	Children (8–11 years)				Adolescents (12–18 years)			
	OR	95% confidence interval		*P*-value	OR	95% confidence interval		*P*-value
1	1				1			
2	2.1	1.5	2.8	<0.001	1.4	1.0	1.9	0.022
3	2.9	2.1	4.0	<0.001	2.4	1.8	3.1	<0.001
≥4	5.0	3.7	6.7	<0.001	4.3	3.4	5.5	<0.001

Adjusted for gender and having chronic illnesses.

## Discussion

High rates of PTSD symptoms among children and adolescents in Jordan were found. Studies in Jordan reveal varying prevalence rates of PTSD between 16.2% and 65.1% (16), likely due to differences in trauma type, population studied, and methodologies. Despite these differences, findings highlight an alarmingly high prevalence, particularly among females and refugees (25–27). PTSD symptoms were higher in adolescents, likely due to the unique characteristics of this developmental stage, with being particularly susceptible to experiencing multiple types of ACEs (28, 29). Adolescence involves biopsychosocial changes, such as evolving relationships and increased reliance on peers, which may lead to risky behaviors and traumatic events, contributing to PTSD vulnerability (30).

The higher rates of PTSD symptom levels in adolescent females than males were consistent with other studies (31, 32). These rates could be explained from different perspectives. Societal norms may encourage females to express emotions, possibly leading to higher reported PTSD rates, while males may underreport. Psychologically, females have a higher tendency for rumination and dwelling on distressing thoughts and feelings, which can exacerbate PTSD symptoms and make females more likely to express greater levels of stress and often internalize traumatic events, contributing to the higher incidence of anxiety, depression, and consequently PTSD. Biologically, hormonal factors like estrogen and differences in brain structures regulating emotions and memory may contribute to this susceptibility (33–36). Another explanation may be that female children and adolescents are at higher risk of certain types of abuse, particularly emotional abuse and psychological maltreatment (37) and female youth exposed to violence exhibit higher trauma symptoms, including PTSD-related symptoms (38).

Children feeling emotionally disregarded by their families had the highest odds of PTSD, consistent with attachment theory. Secure attachment is essential for healthy development, and feelings of neglect can lead to emotional dysregulation and increased PTSD risk (39). Children who feel unloved or emotionally disregarded by their family members often lack sufficient emotional support and guidance in managing stress, impeding their ability to develop effective coping mechanisms, and making them more prone to developing PTSD when exposed to trauma (40–42). Also, the perception of being unloved or unsupported can intensify feelings of isolation and helplessness, which are critical factors in the development of PTSD. Social support is a well-documented protective factor against PTSD, and the absence of perceived familial support significantly increases PTSD risk (43, 44). Findings showed that the sudden accidental death of a family member significantly increased the likelihood of developing PTSD. The sudden and unexpected nature of deaths leaves children particularly vulnerable to PTSD due to the sense of shock and disruption, along with a lack of preparation for the loss (45–47). Studies emphasize the importance of timely and effective psychological interventions to support bereaved young children and adolescents, along with providing targeted mental health care and fostering resilient coping mechanisms (45–47). The higher odds of PTSD in children or adolescents who experience food insecurity and have to wear old clothes suggest a possible association with SES (48).

Exam failure was strongly linked to elevated PTSD symptoms in children. Children encounter significant pressure to perform academically, and failing an exam can be viewed as a major threat to their social standing and self-esteem (7, 49, 50). Unlike adults, coping strategies for children may be inadequately developed to manage such setbacks, making them especially susceptible to stress responses (49). Research indicates that children commonly display intense emotional reactions to academic failures, including shame, fear of punishment, and anxiety about future academic performance (7, 49, 50). COVID-19 infection emerged as the most reported ACE, with stressors like school closures, isolation, and misinformation contributing to PTSD symptoms (51) with findings in line with other studies, highlighting the adverse effects of social isolation and disrupted routines during this pandemic (52, 53).

Research consistently demonstrates that psychological abuse by parents are significantly related to mental health problems in adolescents (54), including PTSD (55) and to extending effects as poor performance in school (56). The long-term effects of severe parental abuse have also been emphasized in the literature, with American psychiatrists studied 156 children who had experienced severe parental abuse and found that 62 (40%) met the diagnostic criteria for PTSD, with 33% continuing to meet the criteria even after two years (57). Moreover, PTSD in these children was associated with internalizing disorders such as anxiety and depression (57).

An increased number of ACE was significantly associated with increased odds of having high PTSD symptoms, in line with the results of previous studies (58–60), which can be explained by various interrelated factors. First, the accumulation of stress overwhelms the development of cognitive and emotional systems among children and adolescents (60). Multiple traumatic experiences increase the psychological burden, which impairs the ability to process and manage stress. Second, exposure to multiple ACEs can adversely impact crucial developmental processes, such as emotional regulation, self-concept, and forming secure attachments (61). These disruptions may reduce children's ability to develop resilience and healthy coping mechanisms, which ultimately increases the odds of high PTSD symptoms (61, 62). Last, each additional traumatic event can intensify or exacerbate the psychological effect of previous traumas (62, 63). A comprehensive approach tailored to the specific needs of adolescents is therefore needed and healthcare professionals should focus on assessing the full spectrum of traumatic experiences, rather than concentrating solely on the most prominent events.

## Policy implications

The high prevalence of PTSD and ACEs among children in Jordan requires urgent attention. Evidence-based interventions to prevent ACE and reduce its impact encompass a range of approaches, including community-wide interventions, parenting programs, home visits, economic and social service interventions, psychological therapies, and school-based programs (64). In Jordan, school-based programs emerge as a suitable option due to the limited accessibility and societal acceptance of psychological therapies, which also applies to other interventions such as parenting programs and home visits. Additionally, school-based programs are supported by evidence for cost-effectiveness (65).

Interventions should adopt a holistic approach that acknowledges the cumulative and collective burden of multiple ACEs, rather than addressing them as isolated events, along with accounting for the broader socioeconomic and environmental factors contributing to adverse experiences (66). The root causes of ACEs extend beyond parenting practices and are also linked to socioeconomic disparities and systemic inequalities. For example, the education ban on females in Afghanistan was found to correlate with a wide range of negative mental health outcomes among females (67). This highlights the necessity of strong social welfare programs, mental health support systems, and other components to create a protective environment that mitigates childhood trauma in a multi-layered approach.

## Limitations

One limitation of this study is its cross-sectional design, which identifies correlations but does not establish causal relationships. Another limitation is the potential for underreporting bias, particularly in sensitive mental health-related topics such as childhood experience. Sensitive issues such as emotional abuse and neglect may be especially susceptible to underreporting, particularly in cultural contexts where stigma, limited awareness, or the tendency to present a socially desirable family image influence response. This potential underreporting may contribute to the underestimation of both the prevalence of ACE and its impact on PTSD symptom levels.

Future research would benefit from employing longitudinal designs to assess the impact of ACEs on mental health outcomes, including PTSD, with stronger causal evidence. Additionally, further studies are encouraged to focus on developing and evaluating intervention programs to determine their effectiveness in addressing ACEs and PTSD.

## Conclusion

The findings highlight a high prevalence of PTSD among children and adolescents exposed to ACEs, with multiple ACEs being associated with high levels of PTSD symptoms and a cumulative pattern of ACEs further increasing the likelihood of experiencing high PTSD symptoms levels. These results underscore the need for targeted interventions that address key ACEs in specific age groups and the development of holistic and comprehensive prevention programs that integrate support for parents, families, schools, communities, and broader societal contexts. In addition, interventions should be designed to mitigate the cumulative and collective effects of multiple ACEs and address different ACEs simultaneously rather than in isolation.

## Data Availability

The raw data supporting the conclusions of this article will be made available by the authors, without undue reservation.
